# An Ultradian Feeding Schedule in Rats Affects Metabolic Gene Expression in Liver, Brown Adipose Tissue and Skeletal Muscle with Only Mild Effects on Circadian Clocks

**DOI:** 10.3390/ijms19103171

**Published:** 2018-10-15

**Authors:** Paul de Goede, Satish Sen, Yan Su, Ewout Foppen, Vincent-Joseph Poirel, Etienne Challet, Andries Kalsbeek

**Affiliations:** 1 Laboratory of Endocrinology, Department of Clinical Chemistry, Amsterdam UMC, University of Amsterdam, Amsterdam 1105 AZ, The Netherlands; p.de.goede@nin.knaw.nl (P.d.G.); ssenbiotech14@gmail.com (S.S.); yan.su@gu.se (Y.S.); e.foppen@nin.knaw.nl (E.F.); 2 Hypothalamic Integration Mechanisms Group, Netherlands Institute for Neuroscience (NIN), Amsterdam 1105 BA, The Netherlands; 3 Circadian Clocks & Metabolism Team, Institute of Cellular and Integrative Neurosciences, UPR3212, Centre National de la Recherche Scientifique (CNRS), University of Strasbourg, Strasbourg 67000, France; challet@inci-cnrs.unistra.fr; 4 Melatonin and Seasonal Rhythms Team, Institute of Cellular and Integrative Neurosciences, UPR3212, Centre National de la Recherche Scientifique (CNRS), University of Strasbourg, Strasbourg 67000, France; vjpoirel@inci-cnrs.unistra.fr; 5 Department of Endocrinology and Metabolism, Amsterdam UMC, University of Amsterdam, Amsterdam 1105 AZ, The Netherlands

**Keywords:** suprachiasmatic nucleus (SCN), circadian clock, soleus muscle (SM), brown adipose tissue (BAT), liver, 6-meal feeding, respiratory exchange ratio (RER), clock genes, metabolic genes, shift work

## Abstract

Restricted feeding is well known to affect expression profiles of both clock and metabolic genes. However, it is unknown whether these changes in metabolic gene expression result from changes in the molecular clock or in feeding behavior. Here we eliminated the daily rhythm in feeding behavior by providing 6 meals evenly distributed over the light/dark-cycle. Animals on this 6-meals-a-day feeding schedule retained the normal day/night difference in physiological parameters including body temperature and locomotor activity. The daily rhythm in respiratory exchange ratio (RER), however, was significantly phase-shifted through increased utilization of carbohydrates during the light phase and increased lipid oxidation during the dark phase. This 6-meals-a-day feeding schedule did not have a major impact on the clock gene expression rhythms in the master clock, but did have mild effects on peripheral clocks. In contrast, genes involved in glucose and lipid metabolism showed differential expression. In conclusion, eliminating the daily rhythm in feeding behavior in rats does not affect the master clock and only mildly affects peripheral clocks, but disturbs metabolic rhythms in liver, skeletal muscle and brown adipose tissue in a tissue-dependent manner. Thereby, a clear daily rhythm in feeding behavior strongly regulates timing of peripheral metabolism, separately from circadian clocks.

## 1. Introduction

Daily recurring biological events in the mammalian behavioral system such as the sleep-wake, feeding-fasting and rest-activity cycles are under the control of the master or central clock, located in the suprachiasmatic nucleus (SCN) in the anterior hypothalamus of the brain. The SCN clock output controls and coordinates other secondary clocks in the brain and peripheral clocks present in virtually every other tissue in the body through neural, humoral and behavioral rhythms, and consequently also regulates bodily rhythms in metabolism [1,2,3,4]. The molecular mechanism underlying the oscillation of this endogenous pacemaker involves a set of clock genes forming transcriptional and (post-)translational feedback circuits [5,6,7]. The positive limb of this molecular clock consists of the core clock elements BMAL1 and CLOCK. Heterodimerization of these core clock elements drives transcription of the Period (Per1, Per2, and Per3) and Cryptochrome genes (Cry1 and Cry2) [8,9,10,11]. The protein isoforms of PER and CRY act as negative components in the feedback loop that inhibit their own transcription [12]. An auxiliary loop that is also induced by CLOCK:BMAL1 heterodimerization activates the rhythmic transcription of Rev-erbα and Rorα. The transcription of Bmal1 and Clock are under the control of both REV-ERBα and RORα, respectively a transcriptional repressor and activator [13,14,15,16,17]. The daily rhythm produced by this molecular clock mechanism has a period of approx. 24 h (i.e., circadian) and is entrained to the exact 24-h rhythm of the outside world by environmental light perceived by the retina and reaching the SCN via the retino-hypothalamic tract (RHT). The molecular clock uses clock-controlled genes such as Dbp, as well as other transcription factors, as an output to regulate a wide variety of cellular processes, of which many involve metabolic processes [18,19]. As such, the biological clock plays a major role in energy metabolism, not only at a whole-body level, but also at a tissue/cellular level.

Circadian disruption, for example, by performing shift work, can thus be expected to have adverse effects on energy homeostasis. Indeed, shift work in humans, as well as in animal models of shift work, shows disturbances in energy metabolism and an increased risk for metabolic disorders such as obesity and type 2 diabetes (T2DM) (e.g., [20,21,22,23,24]). The exact mechanisms that lead from shift-work and disturbed circadian rhythms to these metabolic disorders are not fully understood, but as a result of the disturbed clock mechanisms, metabolic substrate availability and demand may be out of phase and this imbalance may eventually result in metabolic diseases.

Altered or restricted feeding behavior are well known to affect peripheral clocks [25,26,27,28]. An often-used paradigm to study the effects of food intake on metabolism and the circadian clock mechanism is time-restricted feeding (TRF), during which animals have access to food only during a restricted period of the 24-h day, e.g., only during the active or inactive phase. TRF to the inactive phase is often linked with negative effects on health and metabolism, while TRF to the active phase is linked to positive effects [29]. Many studies, including several from our own group, have found that both core clock genes as well as metabolic genes are clearly affected by TRF in metabolically active tissues such as BAT, white adipose tissue (WAT), SM, and liver [25,27,28,30,31,32]. Unfortunately, with the TRF paradigm, it is difficult to distinguish whether the changes in metabolic gene expression are caused by the altered feeding behavior itself or by the feeding-induced changes in the molecular clock mechanism. To disentangle the effects of the molecular clock and the daily rhythm in feeding behavior on rhythms in metabolic gene expression in peripheral tissues such as liver, SM and BAT, we abolished the daily rhythm in feeding-fasting behavior. To accomplish this, we used the 6-meals-a-day feeding paradigm. Under this paradigm, animals have access to food 6 times every 24 h, spread evenly across the day, i.e., one meal every 4 h, and this for several weeks in a row. This well-established paradigm forces the animals to eat similar amounts of food in each of the 6 sessions of food availability, thereby eliminating the natural day/night difference in food intake [33,34]. To better characterize the effects of the 6-meals-a-day feeding regimen on metabolism, we placed rats in metabolic cages, where we measured several physiological parameters, including RER, body temperature and locomotor activity, while the animals remained on the 6-meals-a-day feeding schedule. Additionally, we investigated changes in daily clock gene and protein expression profiles in the SCN, as well as clock and metabolic gene expression profiles in metabolically active peripheral tissues. Our results indicate that while locomotor activity and body temperature rhythms are largely unchanged, RER and metabolic gene expression profiles of animals on the 6-meals-a-day feeding regimen are clearly affected. Interestingly, contrary to mice, 6-meals-a-day feeding in rats did not affect the SCN clock and only mildly affected the peripheral clock mechanism. On the other hand, ultradian feeding clearly affected metabolic gene expression rhythms, although differently for the 3 tissues investigated here: BAT, SM and liver. Thus, the lack of a daily feeding rhythm had a strong impact on timing of metabolism at a whole body and molecular level, despite minor effects on the clock mechanism.

## 2. Results

### 2.1. Caloric Intake and Body Weight Gain

**Figure 1:**
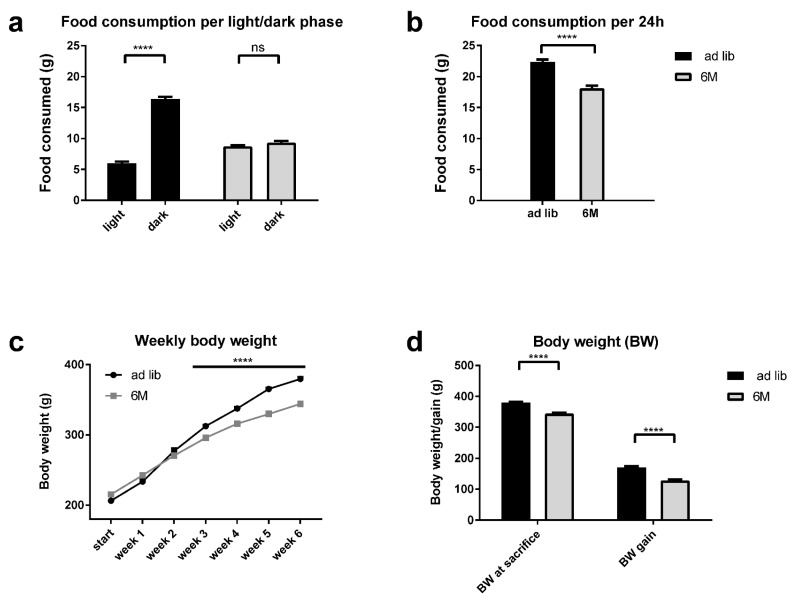
Analysis of the daily food intake and body weight of the subset of animals that have been inside the metabolic cages. (**a**) Difference in daily food intake between light and dark phase for the *ad libitum* and 6M fed animals, i.e., asterisks and ns refer to light/dark differences within the *ad libitum* and 6M group. (**b**) Average 24 h food intake for both groups of animals. (**c**) Growth curve for both groups of animals, animals were weighed on a weekly basis after start of the experiment. (**d**) Body weight at time of sacrifice (**left**) and absolute body weight gain during the experiment (**right**) for both groups of animals. Data are depicted as means ± SEM. ns = non-significant, **** *p* < 0.0001, *n* = 15–16 per group. ad lib = *ad libitum* fed animals (black lines and bars), 6M = animals fed according to the 6 meals a day feeding regimen (grey lines and bars).

Daily caloric intake was lower for the 6-meal group as compared to the *ad libitum* fed group (18.1 g and 22.4 g chow per day (3.1 kcal/g), respectively; independent *t*-test *p* < 0.0001, Figure 1b). Importantly, the L/D difference of food intake seen in *ad libitum* fed animals (73% of food intake during the dark phase; paired *t*-test, *p* < 0.0001) was abolished in the 6-meal group (52% of food intake during the dark phase; paired *t*-test, *p* = 0.075, Figure 1a). Food intake was also not different between the 6 different feeding opportunities in the 6-meal group (one-way ANOVA with repeated measures (RM), *p* = 0.13). Body weight gain after 6 weeks was lower for the 6-meal group as compared to the *ad libitum* fed group (102.2 g and 146.1 g, respectively; independent *t*-test, *p* < 0.0001, Figure 1c,d). However, clearly, all animals in the 6-meal group continued to gain weight throughout the experiment (Figure 1c).

### 2.2. RER

**Figure 2:**
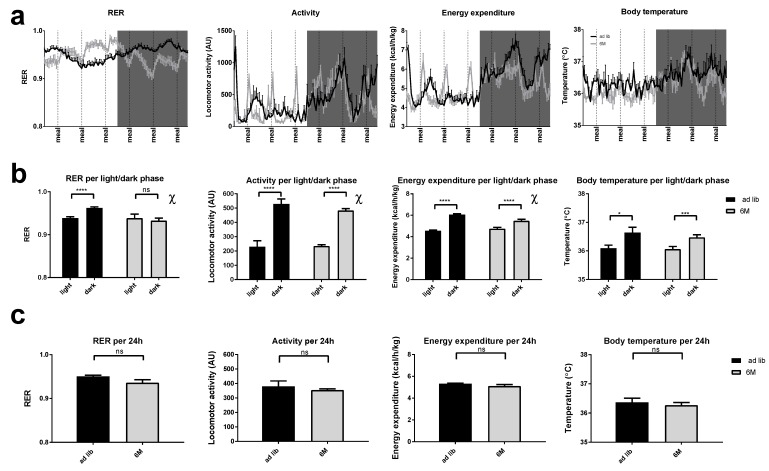
Analysis of the metabolic parameters RER, locomotor activity, heat production and subcutaneous body temperature (from left to right, respectively) of the animals in the metabolic cages. While in the metabolic cages, animals remained on their assigned feeding regimen. (**a**) 24 h traces of the metabolic parameters, averaged per group. The timing of the meals for the 6M group are indicated along the x-axis. (**b**) Difference within metabolic parameters between light and dark phase for the *ad libitum* and 6M fed animals, i.e., asterisks and ns refer to light/dark differences within the *ad libitum* and 6M group. (**c**) Average 24 h values of the metabolic parameters for both groups of animals. Data are depicted as means ± SEM. χ = indicates that a significant interaction was found for Feeding × L/D, ns = non-significant, **** *p* < 0.0001, *** *p* < 0.001, * *p* < 0.05, *n* = 15–16 per group, except for subcutaneous body temperature (*n* = 3–5 per group). Locomotor activity is presented as arbitrary units (AU). ad lib = *ad libitum* fed animals (black lines and bars), 6M = animals fed according to the 6-meals-a-day feeding regimen (grey lines and bars). Shaded areas represent the dark phase.

The daily pattern of RER was altered in the 6-meal group, not showing a clear cosinor-like rhythm alike the *ad libitum* group, but instead 6 peaks in RER throughout the day (Figure 2a). These 6 peaks in RER clearly followed upon the access to food for the 6-meals-a-day group (dotted vertical lines). Nevertheless, cosinor analysis showed a significant daily rhythm for each individual animal in both groups. Further analysis showed that there was a significant L/D difference in RER in the *ad libitum* (paired *t*-test, *p* < 0.0001), but not in the 6-meal animals (paired *t*-test, *p* = 0.354) (Figure 2b). This difference between *ad libitum* and the 6-meals-a-day animals is partly explained by a phase-advance of the daily RER rhythm in the 6 meal group (~ZT20 vs. ZT10; *p* < 0.001) (Table S2). Moreover, the average 24 h RER did not differ between the *ad libitum* and 6 meal groups (unpaired *t*-test, *p* = 0.0581) (Figure 2c). Two-way ANOVA showed significant effects of L/D, as well as Interaction (two way RM-ANOVA; Feeding *p* = 0.055, L/D *p* = 0.008 and Feeding × L/D *p* < 0.001).

### 2.3. Locomotor Activity

The daily pattern of locomotor activity for the 6-meal group was very similar to that of the *ad libitum* group, showing a clear L/D difference, although the 6-meal group also showed 6 peaks in activity over 24 h (Figure 2a). These 6 peaks in locomotor activity mostly followed the access to food for the 6-meal group (dotted vertical lines). Importantly, two-way RM-ANOVA showed that locomotor activity was significantly different between the light and dark period in both *ad libitum* and 6-meal groups, although the significant interaction indicated that this difference was smaller in the 6-meals-a-day group (L/D, *p* < 0.001; Feeding × L/D, *p* = 0.016). A paired *t*-test confirmed the significant effects of L/D in both the *ad libitum* and 6-meal group (*p* < 0.0001 for both groups), with higher locomotor activity during the dark period in both groups (Figure 2b). Cosinor analysis revealed a significant difference in acrophase (2 h) and amplitude of the locomotor activity rhythm between *ad libitum* and 6-meals-a-day feeding, again confirming the larger day/night difference seen in *ad libitum* animals (*p* < 0.001 and *p* = 0.02, respectively) (Table S2). Total locomotor activity per 24 h did not differ between *ad libitum* and the 6-meals-a-day fed rats (independent *t*-test, *p* = 0.538) (Figure 2c). Two-way RM-ANOVA confirmed that locomotor activity per 24 h was not different between *ad libitum* and 6-meals-a-day fed groups (Feeding, *p* = 0.609).

### 2.4. Energy Expenditure

The daily pattern of energy expenditure for the 6-meals-a-day group was similar to the *ad libitum* group, showing a large L/D difference, although the 6-meals-a-day group had 6 clear peaks in energy expenditure throughout the day (Figure 2a). These 6 peaks in energy expenditure were very similar to the peaks in locomotor activity. A subsequent paired *t*-test revealed a statistically significant effect of L/D on energy expenditure in both *ad libitum* and 6-meals-a-day fed rats (*p* < 0.0001), although again with a somewhat smaller amplitude in the 6-meals-a-day animals (Figure 2b). Additionally, the cosinor analysis showed significant differences in the acrophase (2.2 h) and amplitude of energy expenditure between *ad libitum* and 6-meal feeding (*p* < 0.001 and *p* < 0.001, respectively) (Table S2). Energy expenditure per 24 h did not differ between *ad libitum* and 6-meals-a-day fed rats (*p* = 0.136) (Figure 2c). Two-way RM-ANOVA showed a significant effect of L/D (*p* < 0.001) and Feeding × L/D (*p* < 0.001), but no effect of Feeding (*p* = 0.165).

### 2.5. Body Temperature

The daily pattern of body temperature for the 6-meals-a-day group was very similar to that of the *ad libitum* group, showing a clear L/D difference (Figure 2a). Importantly, there were significant differences between the light and dark period, but there was no interaction between Feeding and L/D, nor a main effect of Feeding (Feeding, *p* = 0.214; L/D, *p* = 0.0003; Feeding × L/D, *p* = 0.787; Two-way RM-ANOVA). Cosinor analysis further revealed that there were no significant differences in amplitude or acrophase between the two groups (Table S2). As already confirmed by the two-way RM-ANOVA, total body temperature per 24 h did not differ between *ad libitum* and 6-meals-a-day fed rats (independent *t*-test, *p* = 0.214) (Figure 2c). 

### 2.6. Clock and Clock-Controlled Gene Expression in the SCN

**Figure 3:**
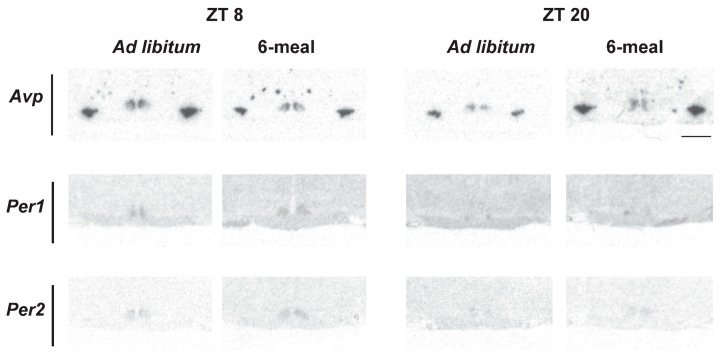
Representative photomicrographs of AVP, Per1 and Per2 expression in the SCN of *ad libitum* and 6-meals-a-day animals at ZT 8 and 20. (Scale bar 1 mm).

**Figure 4:**
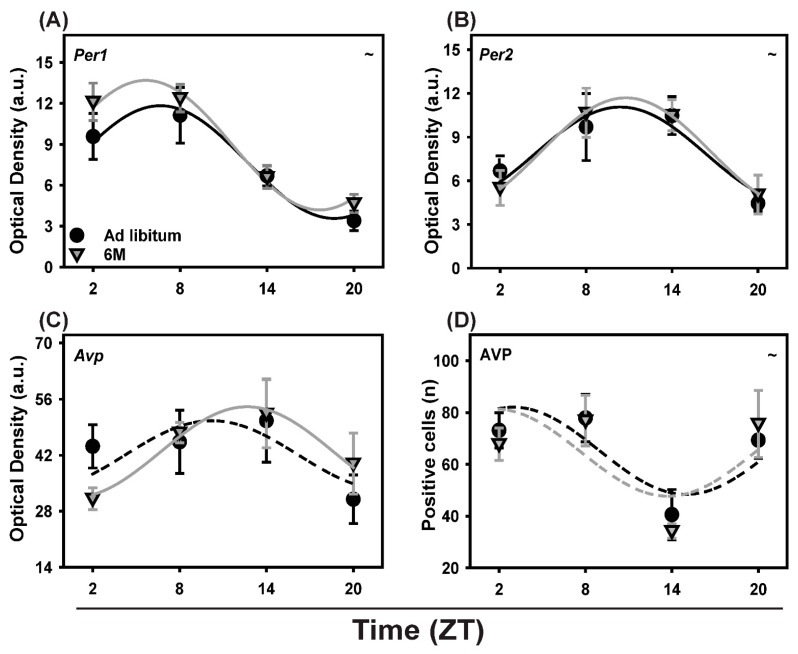
Daily profiles of clock (Per1, Per2) and clock-controlled (AVP) genes and AVP protein expression in the SCN of animals fed *ad libitum* (Black circles) and according the 6-meals-a-day feeding schedule (Gray triangle). (**A**) Per1 mRNA expression; (**B**) Per2 mRNA expression; (**C**) Avp mRNA expression (**D**) AVP protein expression. Fitted lines show significant cosine regressions (see methods). ~ = effect of Time (*p* < 0.05).

The daily pattern of Per1 and Per2 mRNA expression in the SCN was measured under both *ad libitum* and 6-meals-a-day feeding conditions. Two-way ANOVA analysis showed a significant effect of Time for both Per1 and Per2 (*p* < 0.001). Cosinor analysis confirmed the rhythmicity of both clock genes (Figure 3 and Figure 4a,b). Both genes maintained a significant rhythm irrespective of feeding conditions with no significant changes in mean, amplitude or acrophase due to the 6 meals schedule.

The mRNA expression of Avp, a clock-controlled gene, was also examined in the SCN of the *ad libitum* and 6-meals-a-day feeding groups. Photomicrographs of Avp expression in the SCN of *ad libitum* and 6-meals-a-day animals at ZT 8 and ZT 20 are shown in Figure 3. Two-way ANOVA showed no significant effects of Time or Feeding (Table S4). The cosinor analysis detected a significant rhythm in the 6-meals-a-day (*p* = 0.045), but not in the *ad libitum* (*p* = 0.313) group (Figure 4C).

Also, AVP protein expression in the SCN was measured by counting the number of immuno-positive cells in the SCN of rats under *ad libitum* and 6-meals-a-day feeding conditions. Two-way ANOVA showed a significant effect of Time (*p* < 0.001). Cosinor analysis detected no significant rhythmicity in the 6-meals-a-day groups (*p* = 0.139) and a marginally significant rhythm in the *ad libitum* group (*p* = 0.054) (Figure 4D).

### 2.7. Clock Gene Expression in Soleus Muscle

**Figure 5:**
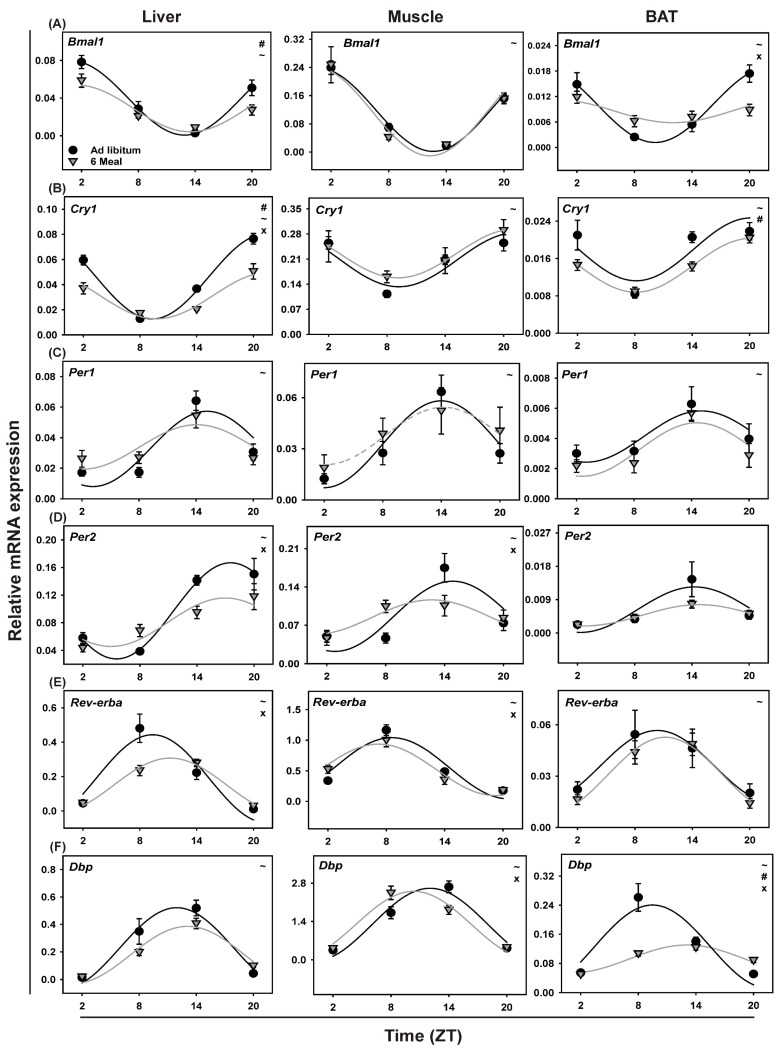
Daily profiles of clock gene expression in Liver, Muscle and BAT in animals fed *ad libitum* (Black circle) or according the 6-meals-a-day feeding schedule (Gray triangle). (**A**) Bmal1, (**B**) Cry1, (**C**) Per1, (**D**) Per2, (**E**) Rev-erbα and (**F**) Dbp. Fitted lines show significant cosine regressions (see methods). ~ = Effect of Time (*p* < 0.05), # = effect of Feeding (*p* < 0.05); × = Interaction effect between Feeding and Time (*p* < 0.05).

Two-way ANOVA analysis showed no significant effect of Feeding for any of the clock genes, but showed a significant effect of Time (*p* < 0.001) for all clock genes except Cry2. A significant interaction between Time and Feeding was found for Per2, Rev-erbα and Dbp (Table 1). All clock genes tested (Bmal1, Per1, Per2, Cry1, Cry2, Rev-erbα and Dbp) (Figure 5A–F, Table 2; cosinor analysis) showed a significant rhythmic expression in the *ad libitum* fed group except Cry2. Eliminating the daily rhythm in feeding behavior with the 6-meals-a-day schedule resulted in a loss of rhythmicity for Per1, while Cry2 remained non-rhythmic. A ~1.5 h shift was observed in Dbp expression, but no other significant changes in mean level or amplitude were observed in the 6-meals-a-day feeding groups (Table 2).

### 2.8. Clock Gene Expression in BAT

All tested clock genes in BAT remained rhythmic in the 6-meals-a-day group except Cry2, which was not rhythmic in either of the feeding conditions (Figure 5, Table 3). Two-way ANOVA showed a significant effect of Time for Bmal1, Cry1, Per2, Dbp and Reverbα. No significant effects of Feeding were found for any of the clock genes studied except for Cry1. A significant Interaction effect for Time and Feeding was found for Bmal1 and Dbp expression (*p* < 0.001). Cosinor analysis revealed no shift in expression for any of the clock genes when comparing *ad libitum* with 6-meals-a-day feeding except for Bmal1 (1.9 h; Table 3).

### 2.9. Clock Gene Expression in Liver

All studied clock genes in the liver remained rhythmic in the 6-meals-a-day feeding conditions, except for Cry2, which lost rhythmicity upon 6-meal feeding. In addition, no significant phase changes were observed except for Rev-erbα (1.9 h; *p* = 0.005). Mean expression levels were affected for Bmal1 and Cry1 and the amplitude was dampened for Bmal1, Cry1, Per2 and Rev-erbα (Table 4). Two-way ANOVA showed an effect of Feeding for Bmal1 and Cry1 (*p* = 0.008 and *p* < 0.001, respectively). Additionally, a significant effect of Time was found for all the studied clock genes as well as an Interaction for Cry1, Per2, and Rev-erbα (Table 1).

### 2.10. Metabolic Genes in Soleus Muscle

**Figure 6:**
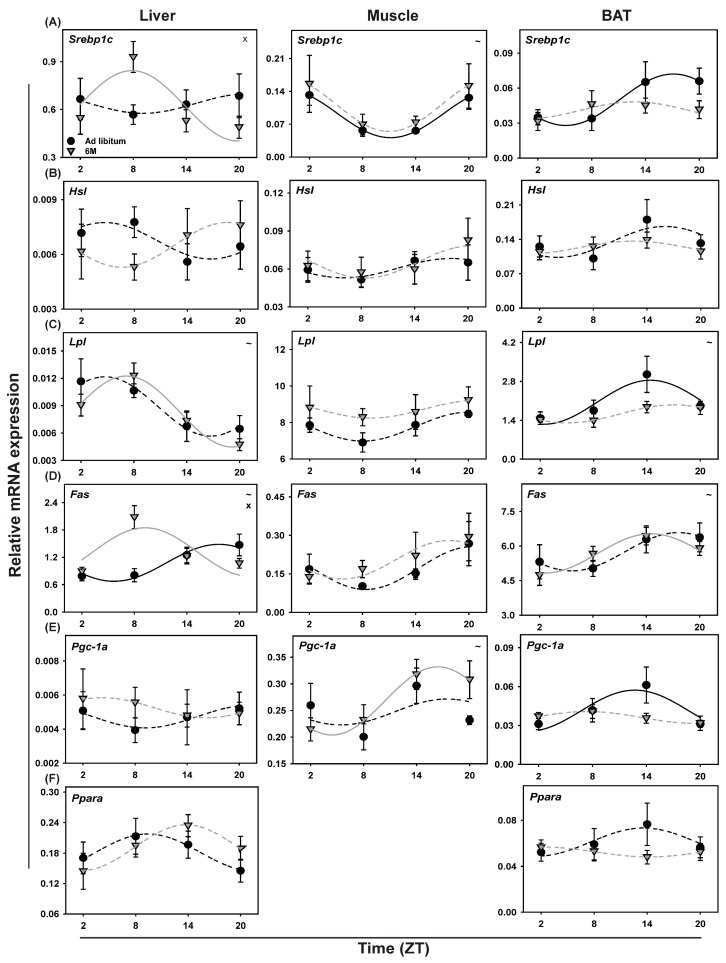

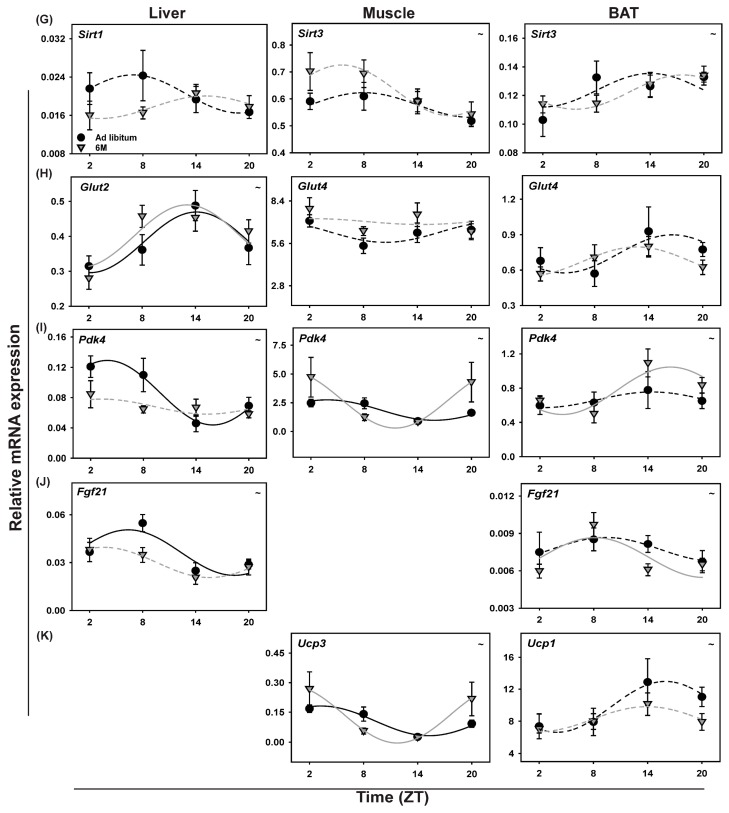
Daily profiles of metabolic gene expression in Liver, Muscle and BAT in animals fed *ad libitum* (Black circles) or according the 6-meals-a-day feeding schedule (Gray triangles). (**A**) Srebp1c, (**B**) Hsl, (**C**) Lpl, (**D**) Fas, (**E**) Pgc-1α, (**F**) Pparα, (**G**) Sirt1 and Sirt3, (**H**) Glut2 and Glut4, (**I**) Pdk4, (**J**) Fgf21 and (**K**) Ucp1 and Ucp3. Fitted lines show significant cosine regressions (see methods). ~ = effect of Time (*p* < 0.05).

Both Pdk4 and Ucp3 showed a shift in peak expression when subjected to the 6-meals-a-day feeding regimen as compared to *ad libitum*. For both genes this shift was in the same direction and of roughly the same magnitude (Figure 6I,K, Table 2). On the other hand, Pgc-1β expression lost rhythmicity under the 6-meal feeding regimen (data not shown). Most of the other metabolic genes studied did not show rhythmicity in either of the feeding conditions except for Srebp1c that lost rhythmicity upon 6-meals-a-day feeding. Pgc1α gained rhythmicity upon 6-meals-a-day feeding (Table 2). Furthermore, two-way ANOVA analysis showed a significant effect of Time for several metabolic genes in soleus muscle (Ucp3, Pgc1α, Pdk4, Srebp1c and Sirt3). No significant effect of Feeding or Interaction was observed for any of the metabolic genes in soleus muscle (Table 1). Out of the 105 possible combinations between the 15 clock and metabolic genes tested, 15 significant correlations in gene expression profile were found (Table S5), with 3 correlations between clock genes, 3 correlations between metabolic genes and 9 correlations between a clock and a metabolic gene. In the 6-meal group 8 of these correlations were maintained, while 7 correlations were lost.

### 2.11. Metabolic Genes in BAT

Cosinor analysis showed loss of rhythmicity for lipid metabolizing genes such as Pgc-1α, Lpl and Srebp1c during 6-meals-a-day feeding, while other genes like Ucp1, Glut4, Pparα, Hsl1 were not rhythmically expressed in either of the two feeding conditions (Figure 6, Table 3). Interestingly, Pdk4, Fas and Fgf21 gained rhythmicity in the 6-meals-a-day fed group as compared to the *ad libitum* group. Two-way ANOVA found no effect of Time in most of the tested metabolic genes, except for Lpl, Pdk4, Ucp1, Sirt3, Fas, and Fgf21. No significant effect of Feeding and Interaction was found for any of the tested metabolic genes in BAT (Table 1). Out of the 136 possible combinations between the 17 clock and metabolic genes tested, 14 significant correlations in gene expression profile were found (Table S5), with 2 correlations between clock genes, 6 correlations between metabolic genes and 6 correlations between a clock and a metabolic gene. In the 6-meal group, only 2 of these correlations were maintained, while 12 correlations were lost. Intriguingly, a significant correlation between Pparα and Per2 was maintained, but for the 6-meal group, a negative correlation between these genes was found, in contrast to the positive correlation found in the *ad libitum* group.

### 2.12. Metabolic Genes in Liver

Cosinor analysis of the metabolic genes tested in the liver showed that upon 6-meals-a-day feeding Glut2 and Fas remained rhythmic. A large phase shift was observed in Fas expression upon 6-meal feeding (8.4 h; *p* = 0.001). Pdk4 and Fgf21 lost rhythmicity, but Lpl and Srebp1c gained rhythmicity. The other metabolic genes tested (Pparα, Pgc-1α, Hsl and Sirt1) were not rhythmic in either condition (Figure 6, Table 4). Two-way ANOVA analysis showed a significant effect of Time for Glut2, Pdk4, Lpl, Fas, and Fgf21. No significant effects of Feeding were found; however, there was a significant Interaction effect for Fas (Table 1). Out of the 121 possible combinations between the 16 clock and metabolic genes tested, 25 significant correlations in gene expression profile were found (Table S5), with 2 correlations between clock genes, 8 correlations between metabolic genes and 15 correlations between a clock and a metabolic gene. In the 6-meal group only 2 of these correlations were maintained, while 23 correlations were lost.

## 3. Discussion

Many studies have investigated the effects of time restricted feeding (TRF) on energy metabolism by focusing on (clock) gene expression in liver, skeletal muscle, BAT and WAT [31,32,35]. Since in these studies clock gene expression profiles were strongly affected by TRF, it was not possible to determine whether the changes in the expression profiles of metabolic genes were driven by the altered rhythms in feeding behavior itself or the altered rhythms in clock gene expression. Additionally, in these studies the animals were fasted for 10–14 h a day, which might also affect metabolism independently of the altered rhythm in feeding behavior. Moreover, disturbed feeding rhythms in humans, including those of shift workers, usually are not characterized by a complete shift of the feeding rhythm, but instead by a more widespread distribution of several smaller “meals” throughout the 24-h day [36,37]. Here we report for the first time on the disruptive effects of an equidistant 6-meals-a-day feeding pattern in rats on SCN, muscle and BAT gene expression rhythms, energy expenditure and RER, as well as confirming our previous findings on body temperature and locomotor activity [38]. This ultradian feeding pattern mainly affected daily rhythms in RER and lipid metabolism, while daily rhythms in clock gene expression, locomotor activity, energy expenditure and body temperature remained relatively intact.

Abolishing the daily feeding-fasting cycle with an ultradian 6-meals-a-day feeding schedule (1 meal every 4 h) without caloric restriction did not affect the SCN clock, and only slightly affected peripheral clock gene expression rhythms in liver, BAT and SM with small changes in acrophase, mesor and amplitude levels for some clock genes in a tissue- and gene-dependent manner. On a whole-body level, minor changes in the daily rhythms in locomotor activity and energy expenditure were found, but the daily rhythm in RER was severely disturbed in the animals on the 6-meals-a-day feeding schedule. In agreement with the disturbed daily RER pattern, eating according to the 6-meals-a-day schedule especially affected expression profiles of genes involved in lipid metabolism in liver, BAT and SM. Hence, in rats, 6-meals-a-day feeding without caloric restriction does not severely impact the molecular clock, but does induce phase changes in the daily rhythmicity of the respiratory quotient, i.e., affect metabolic flexibility.

### 3.1. Ultradian Rhythms in Feeding Behavior Affect Whole Body Metabolism

To successfully eliminate the daily rhythm in feeding behavior we reduced the time of food access, rats could eat ~1.3 h each day evenly spread across the 24-h period. An important issue that arises with this experimental design, especially when investigating metabolism, is that the experimental group eats less and potentially becomes hypocaloric (i.e., losing or not gaining body weight). Importantly, although in our experiments the animals on the 6-meal regimen ate less compared to the *ad libitum* condition, they were not under hypocaloric conditions, as they continuously gained weight throughout the experiment. Nevertheless, at the end of the experiment, body weight was slightly lower when compared to *ad libitum* conditions. This is similar to what was observed in previous studies using the 6-meals-a-day feeding schedule in rats [33,34,39,40]. In contrast, in mice, most of the animals lost weight during such an ultradian 6-meals-a-day feeding regimen [41]. Additionally, in rats, when meal time was reduced to 6 × 8 min, they also became hypocaloric, lost body weight and showed a reduced body temperature [39]. 

During the 6-meals-a-day feeding regimen, the animals largely maintained the day-night rhythm in locomotor activity that is often lost or even inverted to the inactive phase during light phase TRF [27,28]. Despite a small shift in acrophase, the 6-meals rats maintained the normal day-night pattern of locomotor activity even when they are forced to eat during their resting phase, which is consistent with previous reports of 6-meals-a-day feeding in rats. Rats in the present 6-meals-a-day feeding study did not show any anticipatory activity prior to meal access, but a sudden rise in activity was observed during each meal access, especially during the light period. It is known that the SCN controls the daily rhythm in locomotor activity. Since there was no effect of the 6-meals-a-day feeding schedule on the SCN clock, it is not surprising that the animals maintained their regular day-night pattern of locomotor activity and body temperature. On the other hand, when ultradian feeding is combined with caloric restriction, it does affect the SCN clock. For example, alterations in the SCN clock together with changes in locomotor activity and body temperature were found in both rats [39] and mice [41] subjected to a hypocaloric diet with ultradian 6-meals-a-day feeding. The daily RER pattern in the 6-meals-a-day feeding rats did not show the alterations previously observed during daytime TRF [28,32]. In both conditions, the daily RER rhythm showed a profound shift, but in the daytime TRF condition, its amplitude was enhanced, whereas in the 6-meals-a-day feeding condition, if anything, its amplitude was reduced, although this was not significant. This indicates that the length of the daily fasting period is an important determinant of the amplitude of the daily RER rhythm. With TRF the length of the daily fasting period is increased, resulting in more lipid oxidation and a lower RER at the end of the fasting period. On the other hand, with the 6-meals-a-day feeding schedule the maximal fasting period is reduced to 4 h. Surprisingly, despite the very regular feeding period with equal meals and fasting periods, RER still showed a significant daily rhythm, although less regular than in *ad libitum* conditions. Even more surprising, maybe, its acrophase showed a 10-h phase advance, with highest RER values found when they are low in *ad libitum* conditions and lowest values when they are highest during *ad libitum* conditions. These data show that metabolic rhythms and metabolic flexibility may be severely disturbed, even though the circadian system itself is hardly affected.

Energy expenditure in 6-meals-a-day feeding rats showed a sudden rise during each meal access, especially during the light period. This rise in energy expenditure during each meal may be due to meal induced thermogenesis, but it is also strongly correlated with the rise in locomotor activity during each meal access. These rises in activity were most obvious during the light period, i.e., the normal sleep period. Inherently, this paradigm could also potentially alter the sleep/wake cycles of the animals, but this remains to be tested. 

### 3.2. Ultradian Rhythms in Feeding Behavior Do Not Affect the Central SCN Clock

The SCN clock synchronizes the other brain and peripheral clocks. Time-restricted feeding entrains the peripheral clocks, but not the SCN clock. Intriguingly, restricted feeding coupled to caloric restriction does shift main circadian clock output measures (i.e., wheel-running activity and body temperature) in rats and shifts the SCN clock in mice [39,41,42], and so does ultradian feeding coupled with caloric restriction [39,41]. Here, 6-meal feeding in rats did not affect the SCN clock at either the transcriptional or translational level and these findings are consistent with previous behavioral data in non-hypocaloric 6-meal fed rats [39] and other (time-restricted) feeding paradigms [26]. Together, these data make a strong case that the central SCN clock is affected more by the caloric content than the timing of feeding.

### 3.3. Ultradian Feeding Behavior Does Not Dictate the Peripheral Clocks

Peripheral clocks were affected by the 6-meals-a-day feeding paradigm in a tissue-dependent as well as a clock gene dependent manner. For example, rhythmic expression of Per1 in SM and Cry2 in liver was lost under the 6-meals-a-day regimen, while Dbp in BAT (Figure 3) and Dbp and Rev-erbα in eWAT [34] showed a strong reduction in the amplitude of its rhythmic expression. However, most clock genes in liver, SM, BAT and eWAT remained rhythmic under the 6-meals-a-day feeding regimen, clearly indicating that the daily feeding-fasting rhythm is not the only regulator of peripheral clock gene expression. It is likely that the central pacemaker in the SCN, which was found not to be affected by the 6-meals-a-day regimen (Figure 2), also plays a role in the persisting rhythms of these peripheral clocks. Most of the clock genes tested in peripheral tissues, except for Dbp (muscle), Bmal1 (BAT) and Rev-erbα (liver), showed no phase changes which is consistent with the previously reported effect of 6-meals-a-day feeding on the liver [43,44] and eWat [34]. This implies that without a clear day/night difference in food intake, the phase of peripheral clock gene expression is not necessarily affected. In contrast, it has been shown recently that changes in photoperiodic conditions do affect the phase of clock gene expression in the liver when combined with 6-meals-a-day feeding [45]. As rhythms in feeding behavior do not seem to be the only factor dictating daily rhythms in peripheral clock gene expression, clearly other factors such as body temperature, locomotor activity and hormones such as corticosterone and melatonin, which were still found to be rhythmic under the 6-meals-a-day regimen, likely also play a role. Another important difference between TRF experiments and 6-meals-a-day feeding is that during the 6-meals-a-day regimen food deprivation never lasts longer than 4 h, while in TRF studies food access usually is limited to 10–12 h during the light phase, resulting in a daily 12–14 h fasting period. 

### 3.4. Ultradian Feeding Behavior Differentially Affects Metabolic Gene Expression

#### 3.4.1. Soleus Muscle

Under the 6-meals-a-day feeding regimen Srebp1c lost its rhythmic expression, while Pgc1α gained rhythmicity upon 6-meals-a-day feeding. Several metabolic genes also showed a trend for changes on average expression levels as a result of the 6-meal feeding regimen (Glut4, Lpl and Sirt3) or showed a significant phase shift of their acrophase (Pdk4 and Ucp3). This effect of 6-meals-a-day feeding on genes involved in lipid metabolism is in line with our previous observation of increased plasma leptin levels [46]. Finally, the 6-meal feeding regimen appears to affect gene expression profiles in SM less as compared to TRF to the light phase, especially concerning the clock genes [28]. Another interesting notion is that the expression of Sirt3 remained unchanged in muscle, indicating that the 6-meals-a-day feeding schedule did not make the rats calorically restricted. 

#### 3.4.2. BAT

Clock gene expression profiles in BAT and the daily rhythm in RER closely followed the rhythm in feeding behavior during TRF [28], but not with the 6-meals-a-day feeding regimen. Contrasting, several metabolic genes in BAT either lost or gained rhythmicity or had their expression levels altered with 6-meal feeding, including Pdk4, Lpl and Srebp1c, suggesting that glucose and lipid metabolism in BAT is also modulated by 6-meals-a-day feeding schedule. The loss of rhythmicity of Srebp1c, Pgc-1α, and Lpl and gain of rhythmicity of Pdk4, Fgf21, and Fas in BAT during 6-meals-a-day feeding is probably necessary to conserve glucose and lipid metabolism during the active and inactive phase, respectively. Accordingly, when abolishing the day/night rhythm in feeding behavior no L/D difference was observed in RER, although there was a large phase advance resulting in lower RER during the dark phase, suggesting an enhanced utilization of lipids during the dark phase during which the 6-meals-a-day animals were eating less food as compared to the *ad libitum* fed group. As expected, a peak in RER occurred almost immediately after each feeding session in the 6-meals-a-day experiment, regardless of the time of day (Figure 2a). Similar to skeletal muscle, also in BAT, Sirt3 did not show any change in level of expression confirming that during 6-meals-a-day feeding animals were not caloric restricted. Fgf21 is upregulated upon prolonged fasting and stimulates glucose uptake in adipocytes [47,48,49]. Interestingly, average Fgf21 and Pdk4 expression levels remained unchanged during the 6-meals-a-day feeding regimen, but gained rhythmicity. This could suggest that the fasting period is not long enough to induce Fgf21 expression, but still played a role in disturbed glucose uptake as is observed with the gain of rhythmicity of Pdk4 after 6-meals-a-day feeding regimen. Taken together, our 6-meals-a-day data show that in BAT metabolic gene expression is, at least in part, regulated by feeding behavior. On the other hand, clock gene expression in BAT may be more affected by daily food access or fasting period, which is prolonged and more profound in the traditional TRF experiments, but reduced to less than 4 h during our 6-meals-a-day feeding regimen. 

#### 3.4.3. Liver

In the liver, several, but not all, of the metabolic genes were affected by the 6-meals-a-day feeding regimen, but often with opposite effects compared to muscle and BAT. For instance, Srebp1c and Lpl gained rhythmicity with the 6-meals-a-day feeding regimen. Contrasting Pdk4 and Fgf21 lost rhythmicity after 6-meals-a-day feeding, and in these genes, the mean expression levels remained unchanged, suggesting that 4-h fasting is not long enough to activate the expression of Fgf21 and other metabolic genes in the liver (Pparα, Pgc1α, Hsl, and Glut2) that have been found to be activated by prolonged fasting [50,51]. Interestingly, also in the liver, similar to in muscle and BAT, expression of Sirt1 remained unaffected with 6-meals-a-day feeding, indicating again that in the present 6-meal feeding paradigm, the rats were not calorically restricted. Another interesting observation is that Fas expression was completely altered during 6-meal feeding. The different effects on metabolic gene expression in liver when compared to muscle and BAT once more confirms the differential effects of feeding behavior on peripheral metabolically active tissues. Adding to this are the results from the correlation analyses (Table S5), where in the liver 14 out of 15 significant correlations between a clock and metabolic gene were lost upon 6-meal feeding, and for BAT 5 out of 6 of these significant correlations were lost, while for muscle only 4 out of 9 correlations between a clock and a metabolic gene were lost. Although these are correlations only, these results seem to indicate that gene expression in muscle is less affected by the change in feeding pattern, as compared to BAT and liver.

In all three tissue types investigated here, an apparent gain of rhythmicity was found for one or more metabolic genes in the 6-meal group. Although this might seem counter-intuitive, as the rhythm in feeding behavior was abolished, there are several possible explanations for these findings. The natural fasting period of *ad libitum* fed animals during the daytime might disturb the rhythmic expression of these metabolic genes. Elimination of this fasting period through the 6-meal paradigm potentially unmasks this disturbance. In line with this, eliminating the rhythm in feeding behavior only marginally affected the peripheral clocks but did lead to altered expression of metabolic genes. It thus might be possible that the feeding behavior (specifically the daytime fasting period) itself suppressed the rhythmic control of the peripheral clock on these peripheral metabolic genes. This would thus imply that some metabolic genes are mainly regulated by the feeding behavior while other metabolic genes are controlled by the combination of the peripheral clock and feeding/fasting behavior. In line with this is a study with clockless (Cry1/2 KO) mice, showing that for hundreds of metabolic genes in the liver, rhythmic expression could be restored by enforcing TRF [52]. However, a large number of transcripts could not be restored to the same levels and amplitude as those seen in the presence of a functioning circadian oscillator, indicating that feeding behavior and circadian clocks both are necessary to drive rhythmic expression of these peripheral metabolic genes. Finally, the group sizes and the specific ZTs here chosen might lead to insufficient statistical power to detect a significant rhythm in the *ad libitum* group while for the 6-meal group this rhythm is found, for example, through small shifts in the expression profile or a slightly altered amplitude a gene could be detected to be rhythmically expressed in the 6M group, but not in the *ad libitum* group.

Lastly, we do not believe that the duration of our intervention is too short to notice more substantial changes in gene expression profiles and metabolic phenotype. Our study protocol lasted for 6 weeks, and therefore exceeds the duration of most TRF protocols, which generally last between 1 and 4 weeks.

### 3.5. Clinical Relevance of Ultradian and Other TRF Interventions

An increasing amount of evidence suggests that not only diet composition, but also the timing of food intake can both improve or deteriorate energy homeostasis. Recently, several studies in humans have tried to identify potential mechanisms that can explain these links between rhythmic aspects of (feeding) behavior and metabolic diseases, such as obesity and T2DM. One such study found a positive correlation between the fragmentation of daytime activity rhythms and occurrence of obesity and central adiposity in European adolescents [53]. Similar results were found in Spanish obese adult women [54] and Dutch middle-aged and elderly persons [55]. It was hypothesized that this fragmentation of activity was due to circadian disruption, since daily patterns of melatonin, a main output of the SCN, showed a decreased amplitude related to an increase in rhythm fragmentation [54]. When abolishing the feeding-fasting rhythm with our 6-meals-a-day paradigm, the day-night difference in RER was less pronounced in the 6-meals-a-day group. On the other hand, during TRF the amplitude of the daily RER rhythm was enhanced [27,28]. It is, therefore, tempting to speculate that the beneficial effects that are seen during TRF to the active phase result from a clearer distinction between the rest and active phase for physiological parameters such as feeding behavior, energy metabolism and locomotor activity and that this beneficial effect of TRF could also protect against the consequences of an “unhealthy” hypercaloric diet. 

In a different experimental design, mice had access to food twice a day—at the beginning and end of the active period—and several measures of metabolic health were studied [56]. Importantly, in this study, mice in the experimental group consumed equal amounts of food as the *ad libitum* control group. Mice that consumed isocaloric meals twice a day had lower lipid levels, suppressed gluconeogenesis, increased leptin sensitivity, increased muscle mass and decreased adiposity, all suggesting that consuming multiple distinct meals with an equal interval of intermeal fasting, without caloric restriction, can prevent metabolic disorders such as T2DM and obesity. Another possible explanation for these findings is the clear fasting period in the experimental group. Several studies show beneficial effects of (prolonged) fasting on metabolism and health in both humans and rodents (reviewed in [57]). One such study asked overweight, healthy individuals with erratic eating patterns (spread throughout the day and night) to limit their food intake during a 10–12 h period for 16 weeks without reducing caloric intake [37]. After the 16-week intervention period, subjects had reduced body weight, as well as self-reported improved sleep and an improved sense of being energetic. In the same line, mice fed a high-fat diet that were TRF to 8 h during the dark phase (i.e., their usual active period) were protected against obesity, hyperinsulinemia, hepatic steatosis and inflammation as compared to *ad libitum* fed mice that consumed equivalent calories from this high-fat diet [31]. Contrasting, mice fed during the entire 12 h light period showed unfavorable changes in glucose metabolism which can eventually lead to metabolic pathologies [58,59]. However, one study in mice found that when feeding was restricted to 4 h in the light (inactive) phase, this also led to reduced body weight and altered metabolism (lower epididymal fat) when compared to *ad libitum* feeding conditions, similar to studies where food access was restricted to the dark phase [60]. Additionally, cholesterol, HDL, triglyceride, insulin, corticosterone and leptin levels were lower, adiponectin and ghrelin levels were higher and energy markers pAMPK and Sirt1 levels were higher. This would imply that longer daily fasting periods positively affect metabolism regardless of the time of day. Importantly, it should be stressed that in this particular study, the fasted group was compared to *ad libitum* feeding only, and not to a group that was fasted during the dark phase. Furthermore, several studies that compare TRF in both active and inactive phase confirm that the circadian time of food intake has major impact on metabolism, as well as body weight regulation, in addition to the TRF itself [61]. Recently a slightly different TRF experimental design has gained attention: intermittent fasting, during which persons limit their food intake to a short period during the daytime every other day [62,63,64,65]. Another recent study compared eating a conventional 3 meals a day versus 2 meals a day, where either breakfast or dinner was skipped entirely [66]. Even though these 3 different conditions were isocaloric, when a meal was skipped, the 24-h energy expenditure and free fatty acid levels were higher compared to 3 meals a day. In respect to these fasting studies, the 6-meals-a-day feeding protocol has exactly the opposite effect, i.e., it reduces the fasting period to a maximum of 4 h and the animals are continuously in a post-prandial state. Also, human studies that use a higher meal frequency (a conventional 3 versus 6 or even 14 meals during daytime while still allowing one longer fasting period during sleep) showed higher ghrelin levels, as well as increased hunger ratings, hinting that more frequent meals can have a disadvantageous effects on human health [67,68]. Thus, our experimental design not only serves as a valuable tool to study the effects of rhythms in feeding behavior independently of profound changes in the peripheral clocks, but also to study the effects of an absence of a distinct fasting period.

## 4. Materials and Methods

### 4.1. Animal Experiments

Sixty-four male Wistar WU rats, 7 weeks of age upon arrival at the institute (Charles River Breeding Laboratories, Sulzfeld, Germany), were used for the experiments. During the experiment, all animals were housed individually in a controlled climate environment of 21 °C and under 12:12 light:dark conditions for the entire experiment, with Zeitgeber time (ZT) 0 being the time of lights on and ZT12 being the time of lights off. All animals had *ad libitum* access to tap water. Animals only received chow food (Teklad Global Diet, Envigo, Horst, The Netherlands). Animals were habituated to the 6-meals-a-day feeding regimen by having access to their food bin 6 times a day, temporally spaced equally over the 24 h light/dark (L/D)-cycle once every 4 h. In this way there were three feeding opportunities (i.e., meals) during the light period at ZT2, ZT6 and ZT10, each meal consisting of 12 min of food access and three feeding opportunities during the dark period at ZT14, ZT18, and ZT22 each consisting of 11 min of food access, as the animals eat at a slightly higher pace during the dark period. All rats learned within two weeks to eat equal amounts of food in the light and dark period under this feeding regimen. Animals in the control group under *ad libitum* conditions had free access to food 24 h/day. After 4 weeks, a subset of animals from both the 6-meal schedule (*n* = 15) and the *ad libitum* group (*n* = 16) were placed in metabolic cages for 4 days in order to measure physiological and metabolic parameters, while remaining on their assigned feeding conditions. After 6 weeks, the rats were sacrificed at ZT2, ZT8, ZT14 or ZT20 and soleus muscle, liver and BAT tissues were carefully collected, snap frozen in liquid nitrogen and stored at −80 °C until RNA isolation was performed. Brain tissue was collected from two independent experiments with identical experimental procedures. In this way, we could store brain tissue both freshly frozen for use with in situ hybridization as well as store brain tissue post-fixed with 4% paraformaldehyde for immunohistochemistry (IHC). Liver tissue and frozen brain tissue came from one set of animals (*n* = 33), soleus muscle, BAT, PFA fixed brain tissue and metabolic measurements came from the other set of animals (*n* = 31). All experimental procedures were approved by the Animal Ethics Committee of the Royal Dutch Academy of Arts and Sciences (KNAW, Amsterdam, The Netherlands) on 23 September 2010 (Ethic Code: NIN2010.44) and in accordance with the guidelines on animal experimentation of the Netherlands Institute for Neuroscience. 

### 4.2. Activity and Respirometry

Metabolic PhenoCages (TSE systems) were used to measure several metabolic parameters, while animals remained on their *ad libitum* or 6-meals-a-day feeding schedule conditions. After a day of acclimatization to this new environment, the parameters for food intake, locomotor activity, respiratory exchange ratio (RER) and energy expenditure were measured for three consecutive days (72 h). 

### 4.3. Body Temperature

In a subset of animals from both groups, a temperature logger (DST nano-T, STAR ODDI) was placed subcutaneously during a short isoflurane anesthesia (Veterinary Technics, Ijmuiden, The Netherlands) at the lumbar back region in order to prevent interference of the signal with BAT activity. After a recovery period of 9 days body temperature was measured every 15 min for three consecutive days.

### 4.4. RNA Isolation

Soleus muscle tissue was mechanically homogenized while kept on dry ice. The BAT tissue and liver were crushed in Trizol by using a homogenizer machine. For all the tissues, RNA isolation was done using the NucleoSpin RNA isolation kit (Macherey-Nagel, Oensingen, Switzerland). For muscle RNA isolation, three additional washing steps with 75% ethanol were performed. RNA was eluted from the spin column using 40 μL of H2O and RNA concentration and quality of the RNA were determined using a DS-11 (DeNovix, Wilmington, DE, USA) spectrophotometer and a nanochip using Agilent 2100 Bioanalyzer (Agilent Technologies, Santa Clara, CA, USA), respectively. Although RNA integrity number (RIN) values above 5 were considered acceptable, all samples had a RIN above 8.

### 4.5. Muscle, BAT and Liver cDNA Synthesis

Two hundred ng from both muscle and liver, 350 ng from BAT of isolated RNA were used as input template for cDNA synthesis. The Transcriptor First Strand cDNA synthesis kit (Roche, Indianapolis, IN, USA) was used with oligo-dT primers (30 min at 55 °C, 5 min at 85 °C and an additional sample without reverse transcriptase (-RT) was used as negative control to check for genomic DNA contamination during the RT-qPCRs. RT-PCRs were run using an UNO-Thermoblock (Biometra, Jena, Germany).

### 4.6. RT-qPCR

One to nineteen (1:19) diluted cDNA was used for all qPCRs to detect muscle, BAT and liver gene expression profiles. Expression levels of all genes were standardized by dividing over the geometric mean of two or three housekeeping genes: TBP, GAPDH and Cyclophilin for muscle; TBP, HPRT1 and GAPDH for BAT and HPRT and GAPDH for liver. RT-qPCR was performed using a LightCycler 480 (Roche). Different housekeeping genes were used for the different tissues as feeding rhythms are known to affect commonly used housekeeping genes in a tissue-dependent manner and thus need to be tested for each different tissue to determine their suitability [69]. Expression levels were calculated using dedicated software for linear regression of qPCR data (LinRegPCR). All used primers are listed in Table S1. Melting curves of the RT-qPCR and fragment length of the DNA amplicons were inspected as a means of quality control. 

### 4.7. In Situ Hybridization

After decapitation, fresh brains were stored in −80 °C before cryo-sectioning was performed. The transverse sections (20 μm) covering the rostro-caudal axis to the SCN were cut and collected on frosted glass slides in series of 6 sections. Slides were stored at −20 °C until experiments were performed. Antisense and sense RNA probes were generated with an in vitro transcription kit (Maxiscript, Ambion, Austin, TX, USA). Here we used riboprobes of rPer1, rPer2 (plasmids kindly provided by Dr H. Okamura, University of Kyoto, Kyoto, Japan) and rAvp [70]. Hybridization was performed following the protocol described previously [71]. Slides along with a radioactive standard were exposed for 3 days for Avp and 5 days for Per1 and Per2 to an autographic film (Biomax MR Kodak). Standards were included in each cassette to verify that the measured values of optical densities were in the linear response range of the film. Densitometric analysis of hybridization signals was measured using Image J. The optical density of a specific signal was calculated by subtracting the intensity of staining background area measured in an area above the SCN. Measures were taken from the bilateral SCN on five consecutive slices and averaged for the given brain and particular ZT for both feeding conditions. Data were expressed as relative optical density values.

### 4.8. Immunohistochemistry

After decapitation, brains were dissected and post fixed in 4% paraformaldehyde for 24 h in a 4 °C cold room. Following the 24 h post fixation the brain tissue was transferred again to fresh 4% paraformaldehyde for the next 48 h followed by cryoprotection in 30% sucrose solution in a 4 °C cold room until sectioning was performed. Five series of 30 μm coronal SCN sections were prepared on a cryostat and collected in the cryoprotectant and washed with 1× Tris Buffer Saline pH 7.6 (0.1 M TBS). Subsequently, sections were incubated in 3% H_2_O_2_ in TBS (30 min) to suppress endogenous peroxidase activity, thereby reducing background staining. Again, brain sections were rinsed in TBS. Brain sections were then transferred in a solution containing 10% normal serum goat according to the host species of the primary antibody) and Triton X-100 (0.1%) in TBS for 2 h, followed by incubation in the primary antibody (24 h at 4 °C). We used rabbit polyclonal anti arginine-vasopressin (AVP) (1:8000, Truus, a gift from Dr. Ruud Buijs, Netherlands Institute for Brain Research, Amsterdam, The Netherlands). The sections were washed in TBS, then incubated (2 h at 4 °C) with biotinylated goat anti-rabbit IgG (1:500, PK6101; Vectastain Standard Elite ABC Kit Vector Laboratories, Inc., Burlingame, CA, USA) for AVP immunostaining. After this, sections were rinsed in TBS and incubated (2 h) in a solution containing avidin–biotin peroxidase complex (Vectastain Elite ABC kit; Vector Laboratories Inc.). Following incubation with ABC reagents, sections were rinsed 4 times in PBS, and incubated with H_2_O_2_ (0.015%, Sigma-Aldrich, St Louis, MO, USA) and 3,3′-diaminobenzidine tetrahydrochloride (0.5 mg/mL, Sigma-Aldrich) diluted in water. Thereafter, sections were rinsed with TBS, wet mounted on slides coated with gelatin, dehydrated through a series of alcohols, soaked in xylene, and cover slipped. Photomicrographs were taken on a Leica DMRB microscope (Leica Microsystems, Nanterre, France) with an Olympus DP50 digital camera (Olympus, Rungis, France). The number of immune-positive cells was counted on one medial section in both SCN’s and averaged.

### 4.9. Statistics

All data are presented as means ± SEM. Daily rhythmicity of gene and protein expression profiles were assessed using cosinor analysis determining mean, amplitude and acrophase of the considered measures with SigmaPlot 13 software (SPSS Inc, Chicago, IL, USA). Data were fitted to the following regression: y = A + B·cos(2π(x − C)/24), where A is the mean level, B the amplitude and C the acrophase of the rhythm, using SigmaPlot 13 software. Statistical analysis using *t*-tests and two-way analyses of variance (ANOVA) were performed on parameters obtained from cosinor analysis (i.e., A, B and C), and gene and protein expression profiles. Two-way ANOVAs (repeated measures) were performed to assess the effects on the physiological measures (food consumption, weekly body weight gain, RER, locomotor activity, energy expenditure and body temperature) using GraphPad Prism 7. Also (paired) *t*-tests to determine differences between the light and dark phase, as well as differences in 24 h physiological measures between the *ad libitum* and 6M group were performed using GraphPad Prism 7. Finally, Pearson correlations coefficients between the different clock and metabolic genes of the peripheral tissues were also calculated using GraphPad Prism 7.

## 5. Conclusions

Using our 6-meals-a-day feeding regimen to eliminate the daily rhythm in feeding behavior, we observed that this ultradian feeding behavior has no effect on the SCN clock, and has limited effects on peripheral clock gene rhythms in liver, BAT and SM, as long as the animals do not become hypocaloric. These results indicate that other, environmental or endogenous cues clearly also are involved in the control of these peripheral rhythms. The lack of effect of the 6-meals-a-day feeding paradigm on the central pacemaker in the SCN confirms its rigidity to the synchronizing effects of light. However, this does not necessarily prevent misalignment between peripheral clocks and the central clock or between peripheral clocks themselves, because the changes in gene expression were often regulated in a tissue-dependent manner. Finally, the observed changes concerned mainly genes involved in lipid metabolism in either liver or eWAT ([34,41], present study). Together, these results stress the importance of metabolic flexibility and multi-tissue investigations when studying the interrelation of biological rhythms and energy metabolism.

## Figures and Tables

**Table 1:** Two-way ANOVA *p* values for the effect of feeding, time and interaction for the *ad libitum* and 6-meal feeding in liver, Muscle and BAT. Significant effects are indicated in bold.

Genes	Two Way ANOVA Table (*p* Values)								
	Liver			Muscle			BAT		
	Feeding	Time	Interaction	Feeding	Time	Interaction	Feeding	Time	Interaction
*Bmal1*	**=0.008**	**<0.001**	=0.057	=0.758	**<0.001**	=0.830	=0.199	**<0.001**	**=0.002**
*Cry1*	**<0.001**	**<0.001**	**<0.001**	=0.345	**<0.001**	=0.637	**=0.003**	**<0.001**	=0.074
*Cry 2*	=0.270	**=0.021**	=0.516	=0.342	=0.353	=0.670	=0.051	=0.178	=0.684
*Per1*	=0.765	**<0.001**	=0.178	=0.466	**<0.001**	=0.563	=0.117	**<0.001**	=0.991
*Per2*	=0.072	**<0.001**	**=0.021**	=0.888	**<0.001**	**=0.003**	=0.262	**<0.001**	=0.175
*Dbp*	=0.141	**<0.001**	=0.105	=0.969	**<0.001**	**<0.001**	=0.002	**<0.001**	**<0.001**
*Rev-erbα*	=0.122	**<0.001**	**<0.001**	=0.583	**<0.001**	**=0.049**	=0.360	**<0.001**	=0.865
*Ucp1*							=0.166	**=0.039**	=0.654
*Ucp3*				=0.299	**=0.002**	=0.110			
*Pparα*	=0.644	=0.122	=0.459				=0.252	=0.870	=0.471
*Pgc-1α*	=0.526	=0.927	=0.848	=0.340	**=0.031**	=0.271	=0.311	=0.079	=0.121
*Glut2*	=0.505	**<0.001**	=0.234						
*Glut4*				=0.093	=0.066	=0.684	=0.415	=0.126	=0.531
*Pdk4*	=0.063	**=0.004**	=0.082	=0.161	**=0.023**	=0.115	=0.248	**=0.027**	=0.348
*Hsl*	=0.803	=0.954	=0.359	=0.559	=0.427	=0.776	=0.485	=0.187	=0.546
*Lpl*	=0.624	**<0.001**	=0.495	=0.077	=0.144	=0.965	=0.065	**=0.010**	=0.252
*Srebp1c*	=0.840	=0.282	**=0.026**	=0.379	**=0.015**	=0.997	=0.224	=0.110	=0.287
*Sirt1*	=0.194	=0.718	=0.318						
*Sirt3*				=0.104	**=0.045**	=0.606	=0.818	**=0.023**	=0.341
*Fas*	=0.053	**=0.006**	**<0.001**	=0.459	=0.101	=0.832	=0.836	**=0.027**	=0.581
*Fgf21*	=0.098	**<0.001**	=0.181				=0.298	**=0.024**	=0.281

**Table 2:** *t*-Test analysis on cosinor parameters for clock and metabolic genes in the muscle.

Genes		*Ad libitum*		6-Meal		
		Mean	SEM	Mean	SEM	*p* Value
*Bmal1*	a	0.12	0.007	0.11	0.01	=0.767
	b	0.12	0.01	0.13	0.02	=0.719
	c	0.7	0.32	0.3	0.60	=0.595
*Cry1*	a	0.21	0.01	0.22	0.01	=0.351
	b	0.08	0.01	0.07	0.02	=0.788
	c	21.2	0.71	21.1	1.28	=0.953
*Cry2*	a	0.10	0.01	0.12	0.01	=0.335
	b	--	--	--	--	
	c	--	--	--	--	
*Per1*	a	0.03	0.003	0.04	0.01	=0.463
	b	0.03	0.01	--	--	
	c	14.0	0.75	--	--	
*Per2*	a	0.09	0.01	0.09	0.01	=0.899
	b	0.06	0.01	0.03	0.01	=0.074
	c	14.8	0.86	12.7	1.28	=0.169
*Dbp*	a	1.29	0.09	1.28	0.08	=0.973
	b	1.33	0.13	1.22	0.12	=0.548
	c	12.1 ^α^	0.37	10.4	0.38	**=0.002**
*Rev-erbα*	a	0.54	0.04	0.51	0.04	=0.658
	b	0.50	0.06	0.42	0.06	=0.340
	c	8.6	0.44	7.2	0.50	=0.055
*Ucp3*	a	0.10	0.01	0.14	0.03	=0.286
	b	0.07	0.02	0.15	0.04	=0.122
	c	3.3 ^α^	0.80	23.8	1.10	**<0.001**
*Pgc-1α*	a	0.25	0.02	0.27	0.01	=0.355
	b	--	--	0.07	0.02	
	c	--	--	16.4	1.19	
*Glut4*	a	6.33	0.27	7.04	0.33	=0.112
	b	--	--	--	--	
	c	--	--	--	--	
*Pdk4*	a	1.86	0.16	2.78	0.60	=0.148
	b	0.90	0.22	2.48	0.85	=0.077
	c	3.8 ^α^	0.95	23.5	1.30	**<0.001**
*Hsl*	a	0.06	0.01	0.07	0.01	=0.549
	b	--	--	--	--	
	c	--	--	--	--	
*Lpl*	a	7.80	0.24	8.72	0.42	=0.065
	b	--	--	--	--	
	c	--	--	--	--	
*Srebp1c*	a	0.09	0.01	0.11	0.02	=0.364
	b	0.05	0.01	--	--	
	c	23.2	0.92	--	--	
*Sirt3*	a	0.58	0.02	0.63	0.02	=0.096
	b	--	--	--	--	
	c	--	--	--	--	
*Fas*	a	0.17	0.02	0.20	0.03	=0.451
	b	--	--	--	--	
	c	--	--	--	--	

The three fitted parameters of cosinor regressions include a (the mean level), b (the Amplitude), and c (the acrophase of the rhythm; see Methods for details). For the acrophase, the reference time is Zeitgeber 0 (i.e., lights on). ^α^ ad libitum is different from 6-meal (p < 0.05). p values in the right column indicate significance between the ad libitum and 6-meal group. Non-significant parameters are not shown (--). Significant effects are indicated in bold.

**Table 3:** *t*-Test analysis on cosinor parameters for clock and metabolic genes in the BAT.

Genes		*Ad libitum*		6-Meal		*p* Value
		Mean	SEM	Mean	SEM	
*Bmal1*	a	0.01	0.001	0.01	0.001	=0.193
	b	0.01 ^α^	0.001	0.002	0.001	**<0.001**
	c	22.1 ^α^	0.57	0.0	1.41	**<0.001**
*Cry1*	a	0.02 ^α^	0.01	0.01	0.001	**<0.001**
	b	0.01	0.001	0.01	0.001	=0.608
	c	20.1	0.89	20.1	0.48	=0.978
*Cry2*	a	0.01 ^α^	0.001	0.01	0.001	**=0.041**
	b	--	--	--	--	
	c	--	--	--	--	
*Per1*	a	0.004	0.001	0.003	0.001	=0.113
	b	0.001	0.001	0.001	0.001	=0.890
	c	3.0	1.44	2.5	0.96	=0.801
*Per2*	a	0.01	0.001	0.004	0.001	=0.286
	b	0.01	0.001	0.003	0.001	=0.103
	c	14.3	1.12	14.8	0.86	=0.732
*Dbp*	a	0.13	0.01	0.09	0.10	=0.741
	b	0.11 ^α^	0.02	0.03	0.004	**<0.001**
	c	9.5 ^α^	0.55	13.1 ^β^	0.46	**<0.001**
*Rev-erbα*	a	0.04	0.004	0.03	0.002	=0.359
	b	0.02	0.01	0.02	0.003	=0.885
	c	10.3	1.22	11.1	0.62	=0.553
*Ucp1*	a	9.80	0.96	8.24	0.50	=0.159
	b	--	--	--	--	
	c	--	--	--	--	
*Pparα*	a	0.01	0.01	0.05	0.003	=0.247
	b	--	--	--	--	
	c	--	--	--	--	
*Pgc-1α*	a	0.04	0.004	0.03	0.002	=0.313
	b	0.02	0.01	--	--	
	c	12.7	1.47	--	--	
*Glut4*	a	0.74	0.07	0.67	0.04	=0.41
	b	--	--	--	--	
	c	--	--	--	--	
*Pdk4*	a	0.66	0.07	0.77	0.06	=0.250
	b	--	--	0.27	0.08	
	c	--	--	16.5	1.13	
*Hsl*	a	0.13	0.01	0.12	0.01	=0.485
	b	--	--	--	--	
	c	--	--	--	--	
*Lpl*	a	2.05	0.20	1.63	0.10	=0.064
	b	0.80	0.28	--	--	
	c	14.4	1.35	--	--	
*Srebp1c*	a	0.05	0.01	0.04	0.004	=0.219
	b	0.02	0.01	--	--	
	c	17.1	1.44	--	--	
*Sirt3*	a	0.12	0.004	0.12	0.003	=0.826
	b	--	--	--	--	
	c	--	--	--	--	
*Fas*	a	5.75	0.29	5.67	0.18	=0.833
	b	--	--	0.85	0.26	
	c	--	--	14.5	1.15	
*Fgf21*	a	0.01	0.01	0.01	0.001	=0.352
	b	--	--	0.001	0.001	
	c	--	--	8.1	1.28	

The three fitted parameters of cosinor regressions include a (the mean level), b (the Amplitude), and c (the acrophase of the rhythm; see Methods for details). For the acrophase, the reference time is Zeitgeber 0 (i.e., lights on). ^α^ *ad libitum* is different from 6-meal (*p* < 0.05). *p* values on the right column indicate significance of the *t*-test analysis between ad libitum and 6-meal group. Non-significant parameters are not shown (--). Significant effects are indicated in bold.

**Table 4:** *t*-test analysis on cosinor parameters for clock and metabolic genes in the Liver.

Genes		*Ad libitum*		6-Meal		*p* Value
		Mean	SEM	Mean	SEM	
*Bmal1*	a	0.04 ^α^	0.003	0.03	0.003	**<0.001**
	b	0.40 ^α^	0.01	0.02	0.003	**=0.015**
	c	1.0	0.50	1.5	0.52	=0.402
*Cry1*	a	0.05 ^α^	0.001	0.03	0.002	**<0.001**
	b	0.03 ^α^	0.002	0.02	0.003	**<0.001**
	c	21.3	0.26	21.7	0.62	=0.505
*Cry2*	a	0.09	0.01	0.08	0.01	=0.271
	b	0.03	0.01	--	--	
	c	20.3	1.40	--	--	
*Per1*	a	0.03	0.003	0.04	0.004	=0.762
	b	0.03	0.004	0.01	0.01	=0.092
	c	15.2	0.62	14.1	1.25	=0.449
*Per2*	a	0.10	0.01	0.08	0.01	=0.070
	b	0.07 ^α^	0.01	0.04	0.01	**<0.001**
	c	17.6	0.50	17.0	0.95	=0.602
*Dbp*	a	0.23	0.03	0.18	0.01	=0.158
	b	0.30	0.04	0.20	0.02	=0.087
	c	12.0	0.56	13.1	0.40	=0.100
*Rev-erbα*	a	0.19	0.03	0.15	0.01	=0.184
	b	0.25 ^α^	0.04	0.16	0.01	**=0.021**
	c	9.4 ^α^	0.56	11.2	0.32	**=0.005**
*Pparα*	a	0.18	0.01	0.19	0.01	=0.636
	b	--	--	--	--	
	c	--	--	--	--	
*Pgc-1α*	a	0.01	0.001	0.01	0.001	=0.444
	b	--	--	--	--	
	c	--	--	--	--	
*Glut2*	a	0.39	0.02	0.40	0.02	=0.512
	b	0.09	0.02	0.09	0.03	=0.951
	c	14.1	1.30	13.1	1.07	=0.533
*Pdk4*	a	0.09	0.01	0.07	0.01	=0.056
	b	0.04	0.01	--	--	
	c	3.9	0.96	--	--	
*Hsl*	a	0.01	0.001	0.01	0.001	=0.815
	b	--	--	--	--	
	c	--	--	--	--	
*Lpl*	a	0.01	0.001	0.01	0.001	=0.646
	b	--	--	0.004	0.001	
	c	--	--	7.2	0.80	
*Srebp1c*	a	0.64	0.05	0.63	0.05	=0.841
	b	--	--	0.22	0.07	
	c	--	--	7.9	1.10	
*Sirt1*	a	0.02	0.002	0.02	0.001	=0.178
	b	--	--	--	--	
	c	--	--	--	--	
*Fas*	a	1.08	0.08	1.31	0.10	=0.068
	b	0.40	0.11	0.53	0.13	=0.482
	c	17.7 ^α^	1.13	9.2	0.97	**<0.001**
*Fgf21*	a	0.04	0.003	0.03	0.003	=0.103
	b	0.01	0.004	--	--	
	c	6.4	0.99	--	--	

The three fitted parameters of cosinor regressions include a (the mean level), b (the Amplitude), and c (the acrophase of the rhythm; see Methods for details). For the acrophase, the reference time is Zeitgeber 0 (i.e., lights on). ^α^ ad libitum is different from 6-meal (p < 0.05). p values on the right column indicate significance of the t-test analysis between the ad libitum and 6-meal group. Non-significant parameters are not shown (--). Significant effects are indicated in bold.

## Supplementary Materials

The following are available online at http://www.mdpi.com/1422-0067/19/10/3171/s1.
