# Supplementary material for: An Ultradian Feeding Schedule in Rats Affects Metabolic Gene Expression in Liver, Brown Adipose Tissue and Skeletal Muscle with Only Mild Effects on Circadian Clocks
Source: Int J Mol Sci. 2018 Oct 15;19(10):3171. doi: 10.3390/ijms19103171 (PMC6214081; doi:10.3390/ijms19103171)
Supplement: Supplementary file 1 [file ijms-19-03171-s001.zip › ijms-363132-supp-for proof/Supplementary Table S5 BAT correlations overview.pdf]

BAT

ad lib	Bmal1	Cry1	Cry 2	Per1	Per2	Dbp	Rev-erbα	Ucp1	Ucp3	Pparα	Pgc-1α	Glut2	Glut4	Pdk4	Hsl	Lpl	Srebp1c	Sirt1	Sirt3	Fas	Fgf21
**Bmal1**																					
**Cry1**	0.75																				
**Cry 2**	0.09	0.65																			
**Per1**	-0.28	0.37	0.58																		
**Per2**	-0.41	0.26	0.58	0.99																	
**Dbp**	-0.93	-0.93	-0.44	-0.02	0.11																
**Rev-erbα**	-1.00	-0.77	-0.17	0.28	0.41	0.95															
**Ucp1**	-0.05	0.47	0.37	0.93	0.87	-0.17	0.08														
**Ucp3**																					
**Pparα**	-0.58	0.07	0.47	0.95	0.98	0.30	0.58	0.80													
**Pgc-1α**	-0.71	-0.08	0.46	0.86	0.93	0.43	0.70	0.66		0.98											
**Glut2**																					
**Glut4**	0.12	0.71	0.69	0.92	0.86	-0.41	-0.12			0.74	0.61										
**Pdk4**	-0.41	0.23	0.49	0.99	0.99	0.13	0.42	0.91		0.98	0.91		0.85								
**Hsl**	-0.02	0.64	0.79	0.94	0.90	-0.32	0.00	0.86		0.81	0.72		0.98	0.88							
**Lpl**	-0.41	0.24	0.51	0.99	1.00	0.12	0.42	0.90		0.98	0.92		0.85	1.00	0.89						
**Srebp1c**	0.25	0.61	0.25	0.77	0.67	-0.39	-0.20	0.95		0.56	0.38		0.86	0.72	0.75	0.71					
**Sirt1**																					
**Sirt3**	-0.37	-0.39	-0.60	0.30	0.29	0.49	0.46	0.48		0.37	0.30		0.09	0.39	-0.01	0.37	0.47				
**Fas**	0.37	0.72	0.33	0.73	0.62	-0.52	-0.33	0.91		0.49	0.30		0.88	0.66	0.76	0.65	0.99		0.34		
**Fgf21**	-0.96	-0.73	0.02	0.16	0.30	0.88	0.94	-0.14		0.46	0.63		-0.21	0.27	-0.04	0.28	-0.44		0.12	-0.54	

6M	Bmal1	Cry1	Cry 2	Per1	Per2	Dbp	Rev-erbα	Ucp1	Ucp3	Pparα	Pgc-1α	Glut2	Glut4	Pdk4	Hsl	Lpl	Srebp1c	Sirt1	Sirt3	Fas	Fgf21
**Bmal1**																					
**Cry1**	0.44																				
**Cry 2**	0.04	0.73																			
**Per1**	-0.40	0.10	0.75																		
**Per2**	-0.61	0.17	0.74	0.93																	
**Dbp**	-0.93	-0.25	0.30	0.71	0.86																
**Rev-erbα**	-0.80	-0.69	-0.05	0.60	0.59	0.83															
**Ucp1**	-0.61	-0.05	0.63	0.97	0.96	0.86	0.74														
**Ucp3**																					
**Pparα**	0.70	-0.03	-0.64	-0.92	-0.99	-0.92	-0.70	-0.98													
**Pgc-1α**	-0.31	-0.98	-0.83	-0.26	-0.35	0.09	0.55	-0.13		0.21											
**Glut2**																					
**Glut4**	-0.82	-0.36	0.32	0.82	0.86	0.95	0.92	0.93		-0.92	0.19										
**Pdk4**	-0.09	0.50	0.96	0.90	0.84	0.45	0.21	0.80		-0.77	-0.64		0.53								
**Hsl**	-0.68	-0.40	0.33	0.87	0.81	0.85	0.92	0.93		-0.87	0.23		0.97	0.57							
**Lpl**	-0.20	0.69	0.96	0.74	0.84	0.49	0.05	0.68		-0.75	-0.81		0.43	0.90	0.38						
**Srebp1c**	-0.99	-0.29	0.11	0.47	0.70	0.95	0.74	0.67		-0.77	0.15		0.82	0.21	0.67	0.36					
**Sirt1**																					
**Sirt3**	-0.18	0.79	0.86	0.51	0.69	0.39	-0.15	0.48		-0.60	-0.88		0.24	0.73	0.14	0.95	0.34				
**Fas**	-0.77	0.12	0.61	0.80	0.96	0.93	0.60	0.89		-0.97	-0.29		0.85	0.68	0.75	0.77	0.85		0.69		
**Fgf21**	-0.66	-0.72	-0.76	-0.39	-0.20	0.34	0.44	-0.15		0.07	0.72		0.20	-0.69	0.08	-0.55	0.55		-0.44	0.03	

- significant correlation
- significant correlation lost for 6M feeding
- significant correlation for 6M & ad lib in SAME direction
- significant correlation for 6M & ad lib in OPPOSITE direction
