# Supplementary material for: An Ultradian Feeding Schedule in Rats Affects Metabolic Gene Expression in Liver, Brown Adipose Tissue and Skeletal Muscle with Only Mild Effects on Circadian Clocks
Source: Int J Mol Sci. 2018 Oct 15;19(10):3171. doi: 10.3390/ijms19103171 (PMC6214081; doi:10.3390/ijms19103171)
Supplement: Supplementary file 1 [file ijms-19-03171-s001.zip › ijms-363132-supp-for proof/Supplementary Table S5 Liver correlations overview.pdf]

Liver

ad lib

	Bmal1	Cry1	Cry 2	Per1	Per2	Dbp	Rev-erbα	Ucp1	Ucp3	Pparα	Pgc-1α	Glut2	Glut4	Pdk4	Hsl	Lpl	Srebp1c	Sirt1	Sirt3	Fas	Fgf21
**Bmal1**																					
**Cry1**	0.59																				
**Cry 2**	0.33	0.88																			
**Per1**	-0.76	-0.03	0.02																		
**Per2**	-0.35	0.55	0.69	0.74																	
**Dbp**	-0.95	-0.75	-0.59	0.68	0.11																
**Rev-erbα**	-0.58	-0.97	-0.76	-0.06	-0.53	0.69															
**Ucp1**																					
**Ucp3**																					
**Pparα**	-0.60	-0.99	-0.93	0.12	-0.53	0.79	0.92														
**Pgc-1α**	0.52	0.97	0.76	0.12	0.58	-0.64	-1.00			-0.92											
**Glut2**	-0.90	-0.27	-0.13	0.96	0.61	0.84	0.21			0.34	-0.14										
**Glut4**																					
**Pdk4**	0.70	-0.16	-0.35	-0.92	-0.91	-0.51	0.16			0.12	-0.22	-0.88									
**Hsl**	0.49	-0.34	-0.34	-0.93	-0.89	-0.36	0.41			0.25	-0.47	-0.80		0.92							
**Lpl**	0.54	-0.34	-0.60	-0.75	-0.96	-0.29	0.28			0.34	-0.33	-0.70		0.95	0.82						
**Srebp1c**	0.53	0.98	0.81	0.10	0.60	-0.66	-1.00			-0.94	1.00	-0.16		-0.23	-0.46	-0.36					
**Sirt1**	0.00	-0.80	-0.88	-0.48	-0.94	0.25	0.76			0.79	-0.80	-0.30		0.71	0.75	0.83	-0.82				
**Sirt3**																					
**Fas**	-0.31	0.55	0.78	0.59	0.97	0.03	-0.46			-0.56	0.51	0.50		-0.85	-0.76	-0.97	0.54	-0.93			
**Fgf21**	0.11	-0.67	-0.56	-0.72	-0.88	0.02	0.74			0.57	-0.78	-0.51		0.74	0.92	0.72	-0.76	0.87		-0.75	

6M

	Bmal1	Cry1	Cry 2	Per1	Per2	Dbp	Rev-erbα	Ucp1	Ucp3	Pparα	Pgc-1α	Glut2	Glut4	Pdk4	Hsl	Lpl	Srebp1c	Sirt1	Sirt3	Fas	Fgf21
**Bmal1**																					
**Cry1**	0.48																				
**Cry 2**	-0.16	0.55																			
**Per1**	-0.65	-0.49	0.46																		
**Per2**	-0.66	0.34	0.65	0.29																	
**Dbp**	-0.88	-0.67	0.20	0.91	0.37																
**Rev-erbα**	-0.76	-0.93	-0.26	0.70	0.02	0.89															
**Ucp1**																					
**Ucp3**																					
**Pparα**	-0.97	-0.48	0.30	0.80	0.64	0.94	0.77														
**Pgc-1α**	0.75	-0.12	-0.77	-0.65	-0.91	-0.65	-0.26			-0.81											
**Glut2**	-0.97	-0.46	-0.02	0.43	0.63	0.75	0.70			0.88	-0.62										
**Glut4**																					
**Pdk4**	0.78	-0.06	-0.22	-0.11	-0.88	-0.39	-0.22			-0.66	0.71	-0.85									
**Hsl**	-0.21	0.63	0.97	0.34	0.78	0.14	-0.32			0.31	-0.80	0.07		-0.39							
**Lpl**	0.07	-0.74	-0.95	-0.20	-0.73	0.01	0.47			-0.17	0.71	0.04		0.33	-0.99						
**Srebp1c**	-0.20	-0.66	-0.93	-0.29	-0.36	0.07	0.48			0.04	0.48	0.39		-0.13	-0.87	0.89					
**Sirt1**	-0.76	-0.25	0.65	0.93	0.61	0.88	0.56			0.88	-0.88	0.57		-0.41	0.59	-0.46	-0.40				
**Sirt3**																					
**Fas**	-0.50	-0.69	-0.76	-0.09	-0.07	0.31	0.63			0.33	0.17	0.66		-0.40	-0.68	0.74	0.95	-0.14			
**Fgf**	0.78	0.08	-0.72	-0.82	-0.78	-0.79	-0.43			-0.88	0.97	-0.62		0.58	-0.71	0.60	0.44	-0.97		0.15	

- significant correlation
- significant correlation lost for 6M feeding
- significant correlation for 6M & ad lib in SAME direction
- significant correlation for 6M & ad lib in OPPOSITE direction
