# Supplementary material for: An Ultradian Feeding Schedule in Rats Affects Metabolic Gene Expression in Liver, Brown Adipose Tissue and Skeletal Muscle with Only Mild Effects on Circadian Clocks
Source: Int J Mol Sci. 2018 Oct 15;19(10):3171. doi: 10.3390/ijms19103171 (PMC6214081; doi:10.3390/ijms19103171)
Supplement: Supplementary file 1 [file ijms-19-03171-s001.zip › ijms-363132-supp-for proof/Supplementary Table S5 Muscle correlations overview.pdf]

Muscle

ad lib	Bmal1	Cry1	Cry 2	Per1	Per2	Dbp	Rev-erbα	Ucp1	Ucp3	Pparα	Pgc-1α	Glut2	Glut4	Pdk4	Hsl	Lpl	Srebp1c	Sirt1	Sirt3	Fas	Fgf21
**Bmal1**																					
**Cry1**	0.61																				
**Cry 2**	0.04	0.81																			
**Per1**	-0.87	-0.16	0.43																		
**Per2**	-0.67	0.12	0.65	0.95																	
**Dbp**	-0.93	-0.56	-0.03	0.88	0.74																
**Rev-erbα**	-0.49	-0.99	-0.88	0.04	-0.23	0.48															
**Ucp1**																					
**Ucp3**	0.74	-0.08	-0.64	-0.94	-0.94	-0.68	0.23														
**Pparα**																					
**Pgc-1α**	-0.14	0.52	0.77	0.60	0.81	0.36	-0.54		-0.60												
**Glut2**																					
**Glut4**	0.72	0.93	0.65	-0.28	0.03	-0.56	-0.87		0.12		0.56										
**Pdk4**	0.61	-0.25	-0.77	-0.89	-0.94	-0.57	0.39		0.99		-0.69		-0.05								
**Hsl**	-0.09	0.74	0.99	0.53	0.72	0.07	-0.83		-0.73		0.76		0.55	-0.84							
**Lpl**	0.35	0.92	0.90	0.05	0.27	-0.43	-0.97		-0.35		0.45		0.72	-0.49	0.87						
**Srebp1c**	0.92	0.81	0.35	-0.70	-0.48	-0.94	-0.74		0.46		-0.03		0.80	0.32	0.24	0.67					
**Sirt1**																					
**Sirt3**	-0.28	-0.64	-0.60	0.11	0.02	0.53	0.73		0.22		0.05		-0.35	0.31	-0.60	-0.85	-0.61				
**Fas**	0.40	0.79	0.69	-0.15	0.00	-0.59	-0.85		-0.18		0.09		0.54	-0.30	0.67	0.93	0.72		-0.98		
**Fgf21**																					

6M	Bmal1	Cry1	Cry 2	Per1	Per2	Dbp	Rev-erbα	Ucp1	Ucp3	Pparα	Pgc-1α	Glut2	Glut4	Pdk4	Hsl	Lpl	Srebp1c	Sirt1	Sirt3	Fas	Fgf21
**Bmal1**																					
**Cry1**	0.66																				
**Cry 2**	0.65	0.90																			
**Per1**	-0.88	-0.24	-0.32																		
**Per2**	-0.98	-0.56	-0.49	0.91																	
**Dbp**	-0.88	-0.93	-0.82	0.55	0.82																
**Rev-erbα**	-0.28	-0.85	-0.59	-0.20	0.22	0.69															
**Ucp1**																					
**Ucp3**	0.98	0.77	0.79	-0.80	-0.92	-0.92	-0.37														
**Pparα**																					
**Pgc-1α**	-0.47	0.33	0.15	0.83	0.53	-0.01	-0.71		-0.34												
**Glut2**																					
**Glut4**	0.38	0.04	-0.31	-0.37	-0.53	-0.28	-0.18		0.20		-0.22										
**Pdk4**	0.96	0.82	0.84	-0.74	-0.88	-0.94	-0.43		1.00		-0.27		0.15								
**Hsl**	0.37	0.88	0.94	0.03	-0.20	-0.68	-0.71		0.55		0.47		-0.43	0.62							
**Lpl**	0.60	0.99	0.93	-0.17	-0.48	-0.89	-0.84		0.73		0.38		-0.07	0.79	0.93						
**Srebp1c**	0.93	0.88	0.86	-0.67	-0.86	-0.98	-0.54		0.98		-0.15		0.18	0.99	0.67	0.85					
**Sirt1**																					
**Sirt3**	0.22	-0.58	-0.46	-0.65	-0.34	0.26	0.81		0.06		-0.94		0.34	-0.03	-0.74	-0.64	-0.13				
**Fas**	-0.21	0.57	0.56	0.60	0.36	-0.24	-0.70		-0.02		0.86		-0.55	0.07	0.82	0.64	0.15		-0.97		
**Fgf21**																					

- (green) significant correlation
- (red) significant correlation lost for 6M feeding
- (blue) significant correlation for 6M & ad lib in SAME direction
- (yellow) significant correlation for 6M & ad lib in OPPOSITE direction
