# Supplementary material for: An Ultradian Feeding Schedule in Rats Affects Metabolic Gene Expression in Liver, Brown Adipose Tissue and Skeletal Muscle with Only Mild Effects on Circadian Clocks
Source: Int J Mol Sci. 2018 Oct 15;19(10):3171. doi: 10.3390/ijms19103171 (PMC6214081; doi:10.3390/ijms19103171)
Supplement: Supplementary file 1 [file ijms-19-03171-s001.zip › ijms-363132-supp-for proof/Supplementary Tables S1-S4.pdf]

Table S1: Primer list

Genes	Forward primer	Reverse primer
**Housekeeping**		
Cyclophillin	**ATGTGGTCTTTGGGAAGGTG**	**GAAGGAATGGTTTGATGGGT**
GAPDH	**TGAACGGGAAGCTCACTGG**	**TCCACCACCCTGTTG CTGTA**
HPRT1	**GCAGTACAGCCCCAAAATGG**	**AACAAAGTCTGGCCTGTATCCAA**
TBP	**TTCGTGCCAGAAATGCTGAA**	**TGCACACCATTTTCCCAGAAC**
**Clock**		
Bmal1	**CCGATGACGAACTGAAACACCT**	**TGCAGTGTCCGAGGAAGATAGC**
Cry1	**AAGTCATCGTGCGCATTTCA**	**TCATCATGGTCGTCGGACAGA**
Cry2	**TGGATAAGCACTTGGAACGGAA**	**TGTACAAGTCCCACAGGCGGTA**
DBP	**CCTTTGAACCTGATCCGGCT**	**TGCCTTCTTCATGATTGGCTG**
Per1	**CGCACTTCGGGAGCTCAAACTTC**	**GTCCATGGCACAGGGCTCACC**
Per2	**CACCCTGAAAAGAAAGTGCGA**	**CAACGCCAAGGAGCTCAAGT**
Rev-erbα	**ACAGCTGACACCACCCAGATC**	**CATGGGCATAGGTGAAGATTTCT**
**Metabolic**		
Fas	**CTTGGGTGCCGATTACAACC**	**GCCCTCCCGTACACTCACTC**
Glut2	**GTCCAGAAAGCCCCAGATACC**	**TGCCCCTTAGTCTTTTCAAGCT**
Glut4	**GGGCTGTGAGTGAGTGCTTTC**	**CAGCGAGGCAAGGCTAGA**
HSL	**CACACAGCATGGATTTACGCA**	**ACCTGCAAAGACGTTGGACAG**
LPL	**CAAAACAACCAGGCCTTCGA**	**AGCAATTCCCCGATGTCCA**
Pdk4	**TGGTTTTGGTTACGGCTTGC**	**TGCCAGTTTCTCCTTCGACA**
Pgc1α	**TGCCATTGTTAAGACCGAG**	**GGTCATTTGGTGACTCTGG**
Pparα	**TCACACAATGCAATCCGTTT**	**GGCCTTGACCTTGTTCATGT**
Srebp1c	**ACAAGATTGTGGAGCTCAAGG**	**TGCGCAAGACAGCAGATTTA**
Ucp1	**AATCAGCTTTGCTTCCCTCA**	**GCTTTGTGCTTGCATTCTGA**
Ucp3	**GCACTGCAGCCTGTTTTGCTGA**	**ATAGTCAGGATGGTACCGAGCA**
Sirt1	**TGTTTCCTGTGGGATACCTGA**	**TGAAGAATGGTCTTGGGTCTTT**
Sirt3	**GACATACGGGCTGACGTGAT**	**AGTCGGGGCACTGATTTCTG**
Fgf21	**ACCGCAGTCCAGAAAGTCTC**	**GGCCTCAGACTGGTACACAT**

Table S2: t-test analysis for behavioral and physiological parameters.

		Ad libitum	SEM	6-meal	SEM	*P* value	Shift
**Locomotor Activity**	**a**	379.7	37.9	358.6	8.9	=0.60	
	**b**	221.5	7.1	193.4	9.7	**=0.02**	
	**c**	19.3 (ZT)	0.2	17.3 (ZT)	0.4	**<0.001**	**~ 2h**
**Respiratory exchange ratio**	**a**	0.95	0.003	0.93	0.01	=0.054	
	**b**	0.0214	0.001	0.0206	0.001	=0.73	
	**c**	19.9 (ZT)	0.1	10.1 (ZT)	0.8	**<0.001**	**~ 10h**
**Temperature**	**a**	36.8	0.1	36.25	0.07	**=0.02**	
	**b**	0.48	0.1	0.34	0.02	=0.46	
	**c**	17.8 (ZT)	0.3	17.4 (ZT)	0.5	=0.56	
**Heat production**	**a**	5.3	0.04	5.1	0.14	=0.16	
	**b**	1.1	0.05	0.6	0.01	**<0.001**	
	**c**	19.1 (ZT)	0.1	16.8 (ZT)	0.4	**<0.001**	**~2h**

Table S3: t-test analysis on significant cosinor parameters for the clock and clock-controlled genes and protein in SCN.

**Genes SCN**		***Ad libitum***		**6-meal**		***P* value**
		**Mean**	**SEM**	**Mean**	**SEM**	
Avp	**a**	42.03	3.85	42.79	2.94	=0.879
	**b**	--	--	11.31	4.15	
	**c**	--	--	12.6	1.40	
Per1	**a**	7.70	0.70	8.94	0.45	=0.15
	**b**	4.12	0.99	4.74	0.70	=0.61
	**c**	6.6	0.9	5.6	0.56	=0.35
Per2	**a**	7.82	0.72	7.94	0.65	=0.90
	**b**	3.24	1.02	3.75	0.92	=0.70
	**c**	10.4	1.2	10.7	0.93	=0.80
**Protein SCN**						
**AVP**	**a**	65.25	4.45	64.54	5.65	=0.92
	**b**	--	--	--	--	
	**c**	--	--	--	--	

Table S4: Two-way ANOVA analysis for gene expression in SCN.

**Genes SCN**	**2 Way ANOVA Table (*P* value)**		
	**Feeding**	**Time**	**Interaction**
Avp	=0.880	=0.069	=0.587
Per1	=0.164	**<0.001**	=0.767
Per2	=0.909	**<0.001**	=0.878
**Protein SCN**			
**AVP**	=0.791	**<0.001**	=0.876
